# Association between Red Blood Cell Distribution Width and Diabetic Retinopathy: A 5-Year Retrospective Case-Control Study

**DOI:** 10.1155/2021/6653969

**Published:** 2021-07-06

**Authors:** Yingbo Ma, Shengjie Li, Aiping Zhang, Yi Ma, Yani Wan, Jianping Han, Wenjun Cao, Gezhi Xu

**Affiliations:** ^1^Department of Clinical Laboratory, Eye and ENT Hospital of Fudan University, Shanghai, China; ^2^Department of Ophthalmology, Eye and ENT Hospital of Fudan University, Shanghai, China; ^3^Shanghai Key Laboratory of Visual Impairment and Restoration, Fudan University, Shanghai, China; ^4^NHC Key Laboratory of Myopia, Fudan University, Shanghai, China

## Abstract

**Purpose:**

Red blood cell distribution width (RDW) has been regarded as an emerging biomarker of the general population and cardiovascular disease. In this study, we aimed to evaluate the association between RDW and diabetic retinopathy (DR).

**Methods:**

This case-control study included 167 patients with DR, 131 patients with diabetes mellitus (DM), and 170 age- and sex-matched healthy controls from April 2014 to May 2019. Demographic data, laboratory parameters, and ocular examinations were collected.

**Results:**

RDW values of the DR group were significantly higher than those of the healthy control (*p* < 0.001) and DM group (*p*=0.002). A similar trend was observed when RDW was compared among the 3 groups with respect to age and gender. Logistic regression analysis has shown the OR of RDW was 3.791 (2.33–6.168; *p* < 0.001) against the control group and was 1.348 (0.997–1.823; *p*=0.047) against the DM group.

**Conclusion:**

RDW values were significantly elevated in DR patients, and an elevated RDW was associated with an increased incidence of DR in patients with DM.

## 1. Introduction

Diabetic retinopathy (DR), one of the most common microvascular complication of diabetes mellitus (DM), has been a leading cause of vision impairment and acquired blindness among working-age adults worldwide [1]. Currently, China, with a population of 1.4 billion people, has the largest number of individuals with diabetes than any other country in the world. The reported prevalence rate of DR is up to 18.45% and imposes a huge burden on public health systems [2]. Thus, more cost-economic and convenient methods assisting in distinguishing DR are needed urgently to reduce DR-related visual loss.

The clinically visible lesions of DR are mainly vascular in nature and it represents a common result of metabolic disorder induced by hyperglycemia along with chronic inflammation causing impairment, increased permeability, and leakage of the retinal vessels in the early stage [3]. Moreover, inflammatory factors play a pivotal role in the process of hypoxia and ischaemia in the retina, which are critical components of the development and progression of DR [4].

Red blood cell distribution width (RDW) is a parameter to assess the heterogeneity of circulating erythrocyte size and is traditionally used in the differential diagnosis of anemia. In recent years, high RDW has emerged as a promising hallmark of increased risk of all-cause in the general population [5]. Furthermore, RDW has been proposed to be related to inflammation and has been recognized as a novel prognostic marker that reflects chronic inflammation in patients with cancer, cardiovascular disease, diabetes mellitus, thyroiditis, irritable bowel syndrome, disc hernia, and so on [6–11]. Koma et al. found increased RDW level was an independent predictor of death in patients with lung cancer (HR = 2.15, 95% CI: 1.04–4.46, *p* < 0.05) [12]. Lappe et al. found RDW was a significant predictor of all-cause mortality in patients with cardiovascular disease after a follow-up in a period of 8.4–15.2 years (HR of highest versus lowest RDW quintile = 1.37; 95% CI: 1.29–1.46) [13].

Moreover, RDW also has clinical value in diabetes and diabetes-associated vascular complications. According to the National Health and Nutrition Examination Survey III, subjects in the highest quintile of RDW had a higher prevalence of diabetes [14]. Al-Kindi et al. identified that RDW was a powerful and independent marker for cardiovascular mortality in diabetics [15]. Besides, RDW was independently associated with microalbuminuria in patients with newly diagnosed T2DM and may be treated as an effective predictive index in the evaluation of diabetes nephropathy [16].

Although several studies have investigated the relationship between RDW and DR, the results have been controversial now. Blaslov et al. reported that high RDW might be a risk factor for the pathogenesis and progression of DR, while Malandrino et al. found no association between RDW and DR [17, 18]. In this study, we reviewed the clinical and laboratory data of inpatients with DR over the past five years to investigate whether high RDW was associated with DR.

## 2. Materials and Methods

### 2.1. Study Population

#### 2.1.1. Study Design

This retrospective study was conducted from April 2014 to May 2019. Patients with DR and DM were enrolled from the inpatient service of the Department of Ophthalmology at Eye and ENT Hospital of Fudan University. Control participants were enrolled from the community from among those who underwent annual health screenings. This study was approved by the ethics committee of Eye and ENT Hospital of Fudan University and was conducted in accordance with the ethical principles described by the Declaration of Helsinki. The details of the study were explained to the patients and written informed consent was obtained from them.

#### 2.1.2. Sample Evaluation

This study was a case-control study, with DR patients in the case group, DM and healthy controls in the control group, and RDW as the main observation factor. The matching principle was that the age difference between the subjects in the case group and the control group was within ±3 years, and the matching ratio was 1 : 1:1. PASS 15 software was used to calculate the sample size. According to reports in the previous literature, we set the DR patients of the RDW (OR = 1.5), *σ*_*x*_ = 1.00, *α* = 0.05, *β* = 0.10 and obtained the sample size of each group was 128. Assuming the drop-out rate was 20%, the sample size required was 160 for each group finally.

### 2.2. Inclusion and Exclusion Criteria

The DR group included participants who (1) underwent refractive status and intraocular pressure (IOP), slit-lamp biomicroscopic examination, and color fundus photography and were definitely diagnosed with DR by an ophthalmologist; (2) were aged over 18 years. The DM group included participants who (1) self-reported DM; (2) were aged over 18 years. The control group included age- and sex-matched healthy participants who underwent physical examinations, including blood tests and ocular examination.

The patients with DR and DM were excluded if they had one or more of the following:

1. Glaucoma, age-related macular, Behcet's disease, uveitis, and bacterial or fungal keratitis
2. Other complications of diabetes, including cardiovascular disease, diabetic neuropathy, diabetic nephropathy, and diabetic foot, identified by previous checkup or examinations conducted in our hospital
3. Type 1 diabetes mellitus, cancer, hematologic disorders, coronary artery disease, or chronic kidney diseases at baseline
4. Self-reported systemic inflammatory disease
5. Missing data

The control group participants were excluded if they had

1. Diabetes
2. Cancer, hematologic disorders, cardiovascular disease, chronic liver disease, chronic kidney diseases
3. Self-reported systemic inflammatory disease
4. Glaucoma, age-related macular, Behcet's disease, uveitis, bacterial, or fungal keratitis at baseline
5. Missing data

**Figure 1:**
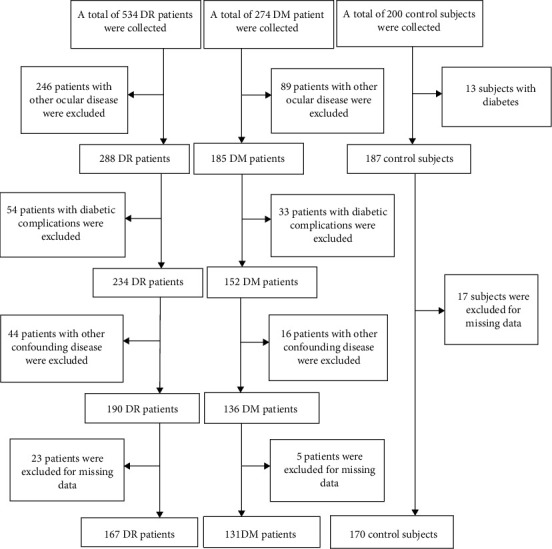
The study population flow chart.

After the exclusion of 540 individuals, a total of 468 age- and sex-matched participants, including 167 patients with DR, 131 patients with DM, and 170 control individuals, were enrolled in the study. The sample size basically met the requirement. The selection process is shown in Figure 1.

### 2.3. Clinical Data and Laboratory Examination

Age, sex, weight, height, hypertension, drinking and smoking status, and disease history were reviewed. Systolic blood pressure (SBP) and diastolic blood pressure (DBP) were measured in millimeters of mercury (mmHg) using a sphygmomanometer after a 5-min rest. Body mass index (BMI) was calculated as weight divided by height squared (kg/m^2^). Each patient with DR and DM underwent detailed ophthalmic examinations, including assessment of refractive status and IOP, slit-lamp biomicroscopic examination, and fundus photography, to assess the presence or absence of retinal ischaemia, clinically significant macular edema, or diabetic retinopathy. Fundus photography was performed with a retinal camera (TRC-NW200, Topcon, Japan). IOP was measured by Goldmann applanation tonometry. The control individuals underwent primary ocular examinations, including assessment of refractive status and IOP and slit-lamp biomicroscopic examination.

In addition, all biochemical analyses were performed at the Department of Clinical Laboratory, Eye and ENT Hospital of Fudan University. 2 mL of peripheral blood samples was routinely collected from all inpatients in ethylenediamine tetraacetic acid (EDTA) tubes and complete blood counts were performed within half an hour of collection. The blood tests included an indicator of white blood cell (WBC), hemoglobin (HG), and RDW; all measurements were performed using a hematology analyzer (Mindray BC5500, Shenzhen, China). Glycated hemoglobin A1c (HbA1c) levels were measured using a glycosylated hemoglobin analyzer (MQ6000, Shanghai, China) within two hours of collecting 2 mL blood samples in EDTA-containing tubes. 4 mL blood samples were collected in coagulant-containing tubes for performing the biochemical estimation of alanine transaminase (ALT), aspartate aminotransferase (AST), blood urea nitrogen (BUN), and creatinine (CRE). The blood samples were centrifuged at 3000 rpm and all tests were performed within two hours of collection using a chemistry analyzer (Roche Cobas, Switzerland). Quality control of the automated analyzers was performed each day before detection. Internal laboratory quality controls were analyzed daily over a 6-year period, without any significant changes in their values (4 < CV < 7%).

### 2.4. Statistical Analyses

The data were analyzed using IBM SPSS 20.0 (Armonk, NY USA). Quantitative variables are expressed as mean ± standard deviation (SD). Kolmogorov–Smirnov test was used to evaluate the distribution of variables. Normally distributed continuous variables were analyzed by independent Student' s *t*-test between two groups or by one-way analysis of variance test among multigroups. For nonnormally distributed data, Mann–Whitney test was used. The difference of rate was tested by Chi-square test and Fisher' s exact probability method, as appropriate. Binary logistic regression analyses were performed to identify the independent risk factors for DM and DR. All reported *p* values were 2-tailed, and those <0.05 were considered to be statistically significant. The Graphpad Prism 8.0 was used for plotting graphs.

## 3. Results

### 3.1. Demographic Characteristics of the Study Population

A total of 167 (45% men and 55% women) patients with DR, 131 (47% men and 53% women) patients with DM, and 170 (50% men and 50% women) healthy controls were enrolled in this study. The demographic characteristics are outlined in Table 1.

There were no significant differences among the control, DM, and DR groups for age, sex, BMI, DBP, hypertension status, and smoking and drinking history (*p* > 0.05). The SBP of DR group was significantly higher than that of the DM and control group (*p* < 0.05, *p* < 0.05). The diabetes duration of the DR group was significantly higher than that of the DM group (*p* < 0.001).

### 3.2. Clinical Data in the DR, DM, and Control Groups

**Figure 2:**
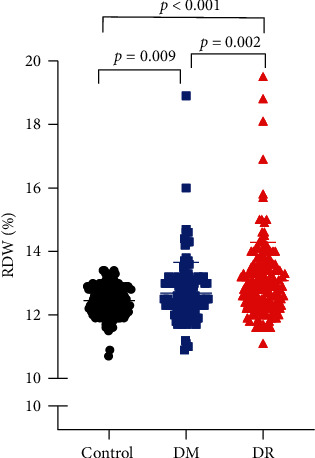
Distribution of RDW among the DR, DM, and control groups.

The mean value of RDW in the DR group (13.09 ± 1.19%) was significantly higher than that in the control (12.45 ± 0.48%) and DM groups (12.68 ± 0.97%) (*p* < 0.001 and *p*=0.002, respectively; Figure 2). The mean value of RDW in the DM group was also significantly higher than that in the control group (*p*=0.009; Figure 2). As displayed in Table 1, the WBC count in the DR group was significantly higher compared with the control and DM groups (*p* < 0.001, *p*=0.002, respectively). The hemoglobin (HG) values in the DR group were significantly lower compared with controls and the DM group (*p* < 0.001, *p* < 0.001, respectively). HbA1c level in the DR group was significantly higher than that in the DM group (*p*=0.039).

### 3.3. Subgroup Analysis of the Study Population

**Figure 3:**
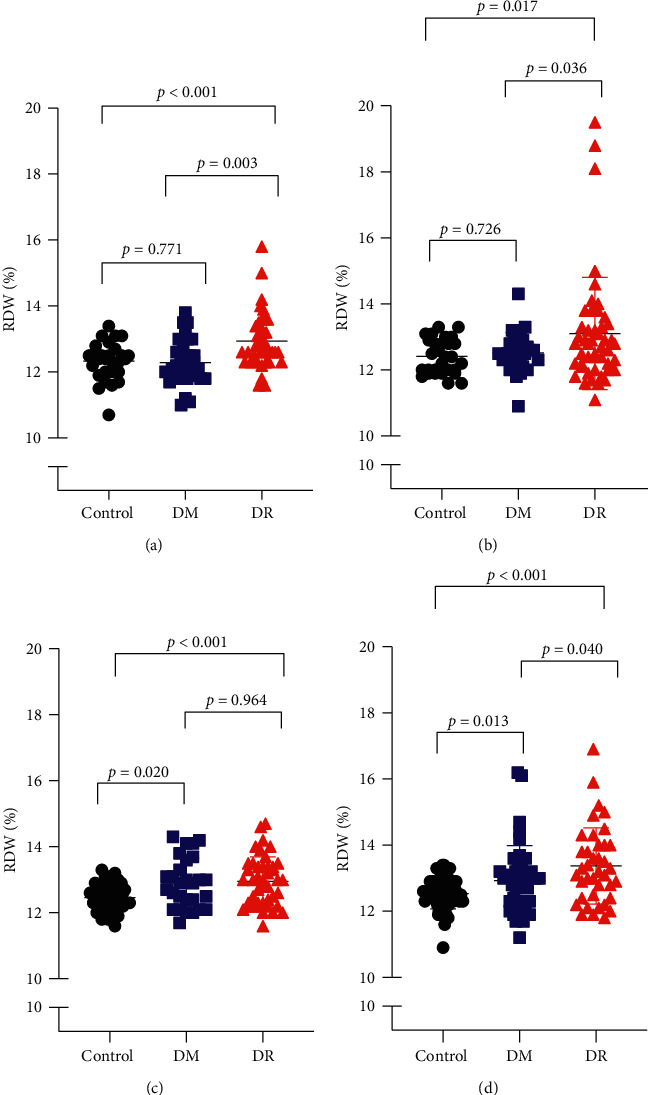
Distribution of RDW among subgroups by age and gender. (a) The distribution of RDW in the men aged under 58. (b) The distribution of RDW in women aged under 58. (c) The distribution of RDW in the men aged over 58. (d) The distribution of RDW in women aged over 58.

The participants were categorized into four subgroups, stratified by sex and the mean age of the DR and DM groups (Table 2). In men under 58 years, the RDW value in the DR group was significantly higher than that in DM and control groups (*p*=0.003, *p* < 0.001; Figure 3(a)). In men over 58 years, the RDW value in the DR group was also significantly higher than DM and control groups (*p*=0.036, *p*=0.017; Figure 3(b)).

Meanwhile, in women under 58 years, the RDW value in the DR group was significantly higher as compared with control group (*p* < 0.001; Figure 3(c)). In women over 58 years, the RDW value in the DR group was significantly higher than the DM and control groups (*p*=0.04, *p* < 0.001; Figure 3(d)).

**Figure 4:**
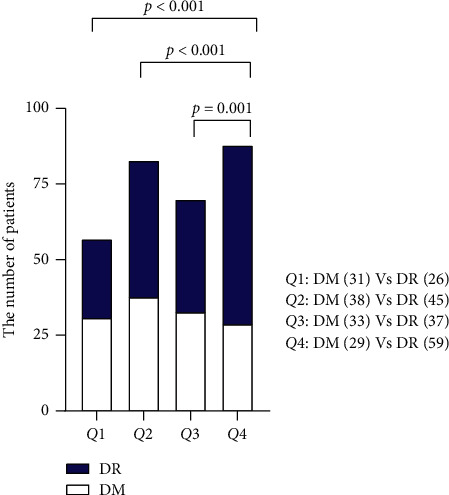
The proportion of DM and DR patients in subgroups defined by the quartile of RDW. *Q*1: 10.9% < RDW ≤ 12.2%, *Q*2: 12.2% < RDW ≤ 12.75%, *Q*3: 12.75% < RDW ≤ 13.3%; and *Q*4: 13.3 < RDW ≤ 19.5%.

Furthermore, the DM and DR patients were divided into four groups according to the quartile of RDW—*Q*1 (10.9%–12.2%), *Q*2 (12.2%–12.75%), *Q*3 (12.75%–13.3%), and *Q*4 (13.3%–19.5%). The number of patients with DM and DR in each group was counted and compared among the 4 quartiles. As showed in Figure 4, the proportion of patients with DR in the *Q*4 group was significantly higher than that in the other groups (vs. *Q*1: *p* < 0.001, vs. *Q*2: *p* < 0.001, and vs. *Q*3: *p* < 0.001).

### 3.4. Correlation Analysis Showing the Relationship between RDW and Other Factors

Pearson correlation analysis showed that age, BMI, SBP, DBP, diabetes duration, WBC, HG, and HbA1c were not associated with RDW (*p* > 0.05; Table 3).

### 3.5. Logistic Regression Analysis Showing the Association between RDW and DR

RDW was identified as a risk factor of DR as shown in Table 4. Against the control group, the OR of RDW was 3.791 (2.330–6.168; *p* < 0.001) after adjusting for age, gender, BMI, and hypertension. SBP and WBC count was also found to be risk factors for DR (*p*=0.004, *p*=0.003) and the OR values were 1.951 (1.039–1.987) and 1.351 (1.106–1.651). Against the DM group, the OR of RDW was 1.348 (0.997–1.823; *p*=0.047) after adjusting for the same confounding factors. HbA1c level and diabetes duration were identified as the other risk factors (*p*=0.019 and *p* < 0.001, respectively). The OR of HbA1c was 1.228 (0.955–1.579). The OR of diabetes duration was 1.178 (1.120–1.240).

## 4. Discussion

In this study, we mainly confirmed the RDW value of patients with DR was significantly higher than that of diabetic patients and controls. Moreover, we identified RDW as a risk factor for DR independent from the traditional risk factors HbA1c and diabetes duration. Limited related literature was found. As far as we are concerned, there are only two papers focusing on the study. Supporting our results, Blaslov et al. performed a prospective cohort study and revealed a significant rise of RDW in DR patients (*p* < 0.001) and stated that it was associated with the risk of DR development and progression (HR = 1.237, *p* < 0.001) [17]. Another cross-sectional study showed the risk of developing DR was not related to the increase of RDW [18]. Considering the complex pathogenesis of DR, it is rational to identify inconsistencies in the results with different study designs and the sample population in different races and duration [19, 20].

Chronic and sustained inflammation plays a critical role in the early alterations that culminate in vascular dysfunction of DR [3]. In the study, we found the significantly increased WBC in DR patients, indicating the inflammatory condition to a certain extent. RDW currently has been considered as an inflammatory marker and reported to be positively correlated with traditional inflammatory biomarkers such as hs-CRP [21, 22]. The possible explanation is that inflammation might lower erythrocyte survival and, in fact, promote anisocytosis, causing immature erythrocytes in larger volumes to enter the blood flow and an increased RDW [23, 24]. In Behcet's disease (BD), a chronic systemic inflammatory disease, Masoumi et al. discovered RDW levels were significantly higher in BD patients with any ocular manifestations and increased RDW level was significantly correlated with the risk of developing ocular diseases in BD patients (OR = 2.031, 95% CI: 1.572–2.625; *p* < 0.001), suggesting that elevated RDW may be related to the ocular vascular inflammation [25]. Aksoy et al. also found an association between RDW and BD ocular activity, supporting the results of Masoumi et al. [26].

In addition, retinal hypoxia and ischaemia are the main triggers of neovascularization and vascular dystrophies of DR, which is key to the progression of DR [27]. In other ocular diseases accompanied by retinal hypoxia, Ozkok et al. found RDW was significantly higher in retinal vein occlusion (RVO) patients (*p* < 0.001) [28]. Pinna et al. also found that RDW was significantly higher in patients with RVO (*p*=0.005) [29]. Besides, they analyzed the correlation between RDW and retinal artery occlusion (RAO) and reported that increased RDW level was associated with increased risk of developing RAO (OR = 1.36, *p*=0.015) [30]. Previous studies revealed that, in the setting of hypoxia, adhesion of RBC to endothelial or subendothelial components was enhanced in microscale blood flow, which could give rise to more fragmentation and hence an elevated RDW [31]. Moreover, it is noteworthy that loss of red cell deformability and enhanced aggregation occur in those patients with DR as well [32]. Such impairment would predispose to red blood cell fragmentation [33]. On the other hand, abnormal deformability and aggregation can also lead to impaired microvascular circulation and hypoxia and ischaemia, worsening the disorder of microcirculation in the retina [34].

Taken together, we speculate that RDW may be associated with DR, but the specific relationship between RDW and disease remains unclear. Based on previous studies on RDW in human diseases, we make the following two conjectures:

1. RDW is a potential biomarker of DR: it is currently believed that the occurrence of DR is accompanied by the inflammatory response, hypoxia, abnormal vascular endothelial function, and so on [35]. These abnormal changes may also lead to impaired red blood cell generation and/or abnormal survival, resulting in increased RDW [24, 36, 37].
2. Elevated RDW is a potential risk factor for DR. The reduced deformability and increased aggregation of RBC in patients with elevated RDW levels will affect the normal ocular microcirculation and oxygen supply, which may be related to the development of DR [38].

The strength of our study was as follows: (1) the patients with DR analyzed in this study were thoroughly free of other macrovascular and microvascular complications such as heart failure, stroke, diabetic foot, or diabetic nephropathy; (2) the critical exclusion criteria mostly eliminated the influence of confounding variables and statistic bias. To our best knowledge, this is the first case-control study focused on the association between RDW and DR patients.

There are also still several limitations of our study. Firstly, it was performed retrospectively. Therefore, we cannot define the causal relationship and its clinical implication as a marker for the progression of DR. Secondly, DR could still be affected by unmeasured additional confounding factors such as subclinical inflammation despite that age, sex, BMI, and hypertension were adjusted.

## 5. Conclusions

In conclusion, our study reports a significantly higher RDW in patients with DR and identifies that increased RDW is an independent risk factor for DR. As a simple, inexpensive and reliable parameter, RDW could make a contribution to assisting in distinguishing DR in the future.

## Figures and Tables

**Table 1:** Demographic characteristics and clinical data of the study population.

	Control (*n* = 170)	Diabetic mellitus (*n* = 131)	Diabetic retinopathy (*n* = 167)	T/F value	*p* value
Age (years)	60.56 ± 9.32	58.50 ± 8.27	58.43 ± 10.96	2.70	0.069
Gender (male/female)	77/93	61/70	85/82	1.146	0.564
BMI (Kg/m^2^)	23.14 ± 3.25	24.50 ± 3.28	24.90 ± 5.10	8.78	**<0.001** ^**a,b,c**^
Smoking (yes/no)	2/168	2/129	3/164	0.384	0.898
Drinking (yes/no)	2/168	4/127	5/162	1.75	0.436
Hypertension (yes/no)	73/97	56/75	81/86	1.30	0.522
SBP (mmHg)	128.58 ± 15.83	136.42 ± 19.79	141.91 ± 37.27	13.04	**<0.001** ^**a,b,c**^
DBP (mmHg)	75.1 ± 9.43	76.48 ± 9.27	76.15 ± 13.40	0.88	0.417
Diabetes duration (years)	—	6.49 ± 5.41	12.52 ± 6.90	8.47	**<0.001**
RDW (%)	12.46 ± 0.48	12.68 ± 0.97	13.09 ± 1.19	20.95	**<0.001** ^**a,b,c**^
WBC (×10^9^)	5.94 ± 1.43	6.14 ± 1.79	6.56 ± 1.80	6.10	**0.003** ^**a,b,c**^
HG (g/L)	135.24 ± 13.17	134.44 ± 13.1	124.43 ± 18.77	20.46	**<0.001** ^**a,b**^
HbA1c (%)	—	6.99 ± 1.31	7.28 ± 1.14	2.08	0.039
AST (U/L)	19.29 ± 7.09	19.76 ± 7.23	20.84 ± 7.87	1.91	0.149
ALT (U/L)	20.19 ± 12.49	22.22 ± 11.09	22.89 ± 11.95	2.32	0.099
BUN (mmol/L)	4.32 ± 0.80	5.86 ± 2.10	6.78 ± 2.61	9.09	**<0.001** ^**a,b,c**^
CRE (*μ*mmol/L)	65.75 ± 14.30	66.17 ± 19.63	71.00 ± 22.32	3.44	**0.034** ^**a,b**^
IOP (mmHg)	12.99 ± 0.21	14.25 ± 0.46	15.19 ± 0.19	13.29	**<0.001** ^**a,b,c**^
VCDR	—	0.32 ± 0.05	0.34 ± 0.07	2.14	**0.030**

Independent sample *t*-test, one-way ANOVA, Chi-square test, and Fisher's exact test were used for analysis. BMI: body mass index, SBP: systolic blood pressure, DBP: diastolic blood pressure. WBC: white blood cell, HG: hemoglobin, HbA1c: glycated hemoglobin A1c, AST: aspartate aminotransferase, ALT: alanine transaminase, BUN: blood urea nitrogen, CRE: creatinine, IOP: intraocular pressure, VCDR: vertical cup-disc ratio. ^a^*p* < 0.05 for the difference between the DR group and the control group (1-way ANOVA with the LSD post hoc test). ^b^*p* < 0.05 for the difference between the DR group and the DM group (1-way ANOVA with the LSD post hoc test). ^c^*p* < 0.05 for the difference between the DM group and the control group (1-way ANOVA with the LSD post hoc test).

**Table 2:** Comparison of RDW by age and gender in DR, DM, and control group.

		Control	Diabetes mellitus	Diabetic retinopathy	F value	*p* value
Male	<=58	12.22 ± 0.58 (*n* = 38)	12.21 ± 0.56 (*n* = 33)	12.95 ± 0.87 (*n* = 37)	11.31	**<0.001** ^a,b^
	>58	12.41 ± 0.53 (*n* = 39)	12.52 ± 0.61 (*n* = 28)	13.11 ± 1.70 (*n* = 48)	3.52	**<0.001** ^a,b^
Female	<=58	12.46 ± 0.42 (*n* = 44)	12.93 ± 0.82 (*n* = 28)	12.95 ± 0.73 (*n* = 43)	7.46	**0.001** ^a,c^
	>58	12.53 ± 0.46 (*n* = 49)	12.99 ± 1.30 (*n* = 42)	13.34 ± 1.11 (*n* = 39)	9.71	**<0.001** ^a,b,c^

Data are expressed as mean ± standard deviation (SD). One-way ANOVA was used. ^a^*p* < 0.05 for the difference between DR group and control group (1-way ANOVA with the LSD post hoc test). ^b^*p* < 0.05 for the difference between DR group and DM group (1-way ANOVA with the LSD post hoc test). ^c^*p* < 0.05 for the difference between DM group and control group (1-way ANOVA with the LSD post hoc test).

**Table 3:** Correlation analysis of RDW with other factors in DR patients.

Variable	*r*	*p* value
Age (year)	0.030	0.703
BMI	−0.005	0.951
SBP (mmHg)	−0.042	0.594
DBP (mmHg)	−0.022	0.775
Diabetes duration (year)	0.049	0.529
WBC (×10^9^)	0.042	0.588
HG (g/L)	−0.063	0.421
HbA1c (%)	0.119	0.126

Pearson correlation analysis was used. BMI: body mass index, SBP: systolic blood pressure, DBP: diastolic blood pressure, WBC: white blood cell, HG: hemoglobin, HbA1c: glycated hemoglobin A1c.

**Table 4:** Logistic regression models evaluating the risk factors in DR.

Variable	Against control group			Against DM group		
	Odds ratio^*∗*^	95% confidence interval	*p* value	Odds ratio^*∗*^	95% confidence interval	*p* value
SBP (mmHg)	1.951	1.039–1.987	0.004	1.007	0.994–1.020	0.269
DBP (mmHg)	0.902	0.919–0.984	0.673	0.987	0.958–1.018	0.410
RDW (%)	**3.791**	**2.330–6.168**	**<0.001**	**1.348**	**0.997–1.823**	**0.047**
WBC (×10^9^)	1.351	1.106–1.651	0.003	1.138	0.968–1.339	0.118
HG (g/L)	0.933	0.912–0.955	<0.001	0.955	0.935–0.975	<0.001
HbA1_c_ (%)				1.228	0.955–1.579	0.019
Diabetes duration (year)				1.178	1.120–1.240	<0.001

Binary logistic regression was used. Odds Ratio^*∗*^: adjusted with age, gender, BMI and hypertension. BMI: body mass index, SBP: systolic blood pressure, DBP: diastolic blood pressure, WBC: white blood cell, HG: hemoglobin, HbA1c: glycated hemoglobin A1c.

## Data Availability

The data used to support the findings of this study are available from the corresponding author upon request.

## References

[1] Cheung N., Mitchell P., Wong T. Y. (2010). Diabetic retinopathy. *The Lancet*.

[2] Song P., Yu J., Chan K. Y., Theodoratou E., Rudan I. (2018). Prevalence, risk factors and burden of diabetic retinopathy in China: a systematic review and meta-analysis. *Journal of Global Health*.

[3] Forrester J. V., Kuffova L., DelibegoviC M. (2020). The role of inflammation in diabetic retinopathy. *Frontiers in Immunology*.

[4] Tayyari F., Khuu L. A., Sivak J. M. (2019). Retinal blood oxygen saturation and aqueous humour biomarkers in early diabetic retinopathy. *Acta Ophthalmologica*.

[5] Pan J., Borné Y., Engström G. (2019). The relationship between red cell distribution width and all-cause and cause-specific mortality in a general population. *Scientific Reports*.

[6] Aktas G., Sit M., Karagoz I. (2017). Could red cell distribution width be a marker of thyroid cancer?. *Journal of College of Physicians and Surgeons Pakistan*.

[7] Chen P.-C., Sung F.-C., Chien K.-L., Hsu H.-C., Su T.-C., Lee Y.-T. (2010). Red blood cell distribution width and risk of cardiovascular events and mortality in a community cohort in Taiwan. *American Journal of Epidemiology*.

[8] Bilgin S., Aktas G., Zahid Kocak M. (2020). Association between novel inflammatory markers derived from hemogram indices and metabolic parameters in type 2 diabetic men. *The Aging Male*.

[9] Aktas G., Sit M., Dikbas O. (2014). Could red cell distribution width be a marker in hashimoto’s thyroiditis?. *Experimental and Clinical Endocrinology and Diabetes*.

[10] Aktas G., Alcelik A., Tekce B. K., Tekelioglu V., Sit M., Savli H. (2014). Red cell distribution width and mean platelet volume in patients with irritable bowel syndrome. *Gastroenterology Review*.

[11] Dagistan Y., Dagistan E., Gezici A. R. (2016). Could red cell distribution width and mean platelet volume be a predictor for lumbar disc hernias?. *Ideggyogy Sz*.

[12] Koma Y., Onishi A., Matsuoka H. (2013). Increased red blood cell distribution width associates with cancer stage and prognosis in patients with lung cancer. *Plos One*.

[13] Lappé J. M., Horne B. D., Shah S. H (2011). Red cell distribution width, C-reactive protein, the complete blood count, and mortality in patients with coronary disease and a normal comparison population. *Clinica Chimica Acta; International Journal of Clinical Chemistry*.

[14] Perlstein T. S., Weuve J., Pfeffer M. A., Beckman J. A. (2009). Red blood cell distribution width and mortality risk in a community-based prospective cohort. *Archives of Internal Medicine*.

[15] Al-kindi S. G., Refaat M., Jayyousi A. (2017). Red cell distribution width is associated with all-cause and cardiovascular mortality in patients with diabetes. *BioMed Research International*.

[16] Zhang M., Zhang Y., Li C., He L. (2015). Association between red blood cell distribution and renal function in patients with untreated type 2 diabetes mellitus. *Renal Failure*.

[17] Blaslov K., Kruljac I., Mirošević G., Gaćina P., Kolonić S. O., Vrkljan M. (2019). The prognostic value of red blood cell characteristics on diabetic retinopathy development and progression in type 2 diabetes mellitus. *Clinical Hemorheology and Microcirculation*.

[18] Malandrino N., Wu W. C., Taveira T. H., Whitlatch H. B., Smith R. J. (2012). Association between red blood cell distribution width and macrovascular and microvascular complications in diabetes. *Diabetologia*.

[19] Loprinzi P. D., Loenneke J. P., Ahmed H. M., Blaha M. J. (2016). Sex and race-ethnicity secular trends in mean and elevated red blood cell distribution width among adults in the United States, 1999–2012. *Ethnicity and Disease*.

[20] Nada A. M. (2015). Red cell distribution width in type 2 diabetic patients. *Diabetes, Metabolic Syndrome and Obesity: Targets and Therapy*.

[21] Krintus M., Kozinski M., Kubica J., Sypniewska G. (2014). Critical appraisal of inflammatory markers in cardiovascular risk stratification. *Critical Reviews in Clinical Laboratory Sciences*.

[22] Lippi G., Targher G., Montagnana M., Salvagno G. L., Zoppini G., Guidi G. C. (2009). Relation between red blood cell distribution width and inflammatory biomarkers in a large cohort of unselected outpatients. *Archives of Pathology and Laboratory Medicine*.

[23] Weiss G., Ganz T., Goodnough L. T. (2019). Anemia of inflammation. *Blood*.

[24] Weiss G., Goodnough L. T. (2005). Anemia of chronic disease. *New England Journal of Medicine*.

[25] Masoumi M., Shadmanfar S., Davatchi F. (2020). Correlation of clinical signs and symptoms of Behçet’s disease with mean platelet volume (MPV) and red cell distribution width (RDW). *Orphanet Journal of Rare Diseases*.

[26] Aksoy Ş. N., Savaş E., Sucu M., Kisacik B., Kul S., Zengin O. (2015). Association between red blood cell distribution width and disease activity in patients with Behçet’s disease. *Journal of International Medical Research*.

[27] Pe’er J., Shweiki D., Itin A. (1995). Hypoxia-induced expression of vascular endothelial growth factor by retinal cells is a common factor in neovascularizing ocular diseases. *Laboratory Investigation*.

[28] Ozkok A., Nesmith B. L. W., Schaal S. (2018). Association of red cell distribution width values with vision potential in retinal vein occlusion. *Ophthalmology Retina*.

[29] Pinna A., Porcu T., Marzano J. (2021). Mean platelet volume, red cell distribution width, and complete blood cell count indices in retinal vein occlusions. *Ophthalmic Epidemiology*.

[30] Pinna A., Porcu T., Paliogiannis P. (2021). Complete blood cell count measures in retinal artery occlusions. *Acta Ophthalmologica*.

[31] Kim M., Alapan Y., Adhikari A. (2017). Hypoxia-enhanced adhesion of red blood cells in microscale flow. *Microcirculation*.

[32] Wautier J.-L., Paton R. C., Wautier M.-P. (1981). Increased adhesion of erythrocytes to endothelial cells in diabetes mellitus and its relation to vascular complications. *New England Journal of Medicine*.

[33] Agrawal R., Smart T., Nobre-cardoso J. (2016). Assessment of red blood cell deformability in type 2 diabetes mellitus and diabetic retinopathy by dual optical tweezers stretching technique. *Scientific Reports*.

[34] Tan J., Wei X., Wong P. A., Fang J., Kim S., Agrawal R. (2020). Altered red blood cell deformability- a novel hypothesis for retinal microangiopathy in diabetic retinopathy. *Microcirculation*.

[35] The Lancet Diabetes and Endocrinology (2020). Under the lens: diabetic retinopathy. *The Lancet Diabetes and Endocrinology*.

[36] Linsenmeier R. A., Braun R. D., Mcripley M. A. (1998). Retinal hypoxia in long-term diabetic cats. *Investigative Ophthalmology and Visual Science*.

[37] Li Y., Zhou Y., Zhang D. (2019). Hypobaric hypoxia regulates iron metabolism in rats. *Journal of Cellular Biochemistry*.

[38] Agrawal R., Sherwood J., Chhablani J. (2016). Red blood cells in retinal vascular disorders. *Blood Cells, Molecules, and Diseases*.

